# Access Deferred Is Access Denied: Small Molecule Drug Pricing and the Future of Dermatology

**DOI:** 10.1111/ijd.70009

**Published:** 2025-08-14

**Authors:** Nikhita J. Perry, John S. Barbieri, Lee A. Fleisher

**Affiliations:** ^1^ Perelman School of Medicine University of Pennsylvania Philadelphia Pennsylvania USA; ^2^ Harvard Medical School Boston Massachusetts USA; ^3^ Department of Dermatology Brigham and Women's Hospital Boston Massachusetts USA; ^4^ Rubrum Advising Philadelphia Pennsylvania USA

**Keywords:** drug pricing, inflation reduction act, small molecule therapies

On April 15, President Trump signed an Executive Order (E.O.) directing the United States Department of Health and Human Services (HHS) to propose legislative changes to the Inflation Reduction Act (IRA), including addressing what some have called the “pill penalty.” Passed in 2022, the IRA allows Medicare, the US federal health insurance program for adults aged 65 and older, to negotiate drug prices with pharmaceutical manufacturers for certain high‐cost medications with the goal of lowering out‐of‐pocket costs and reducing government spending. This authority is notable because, unlike many other high‐income countries, the US government historically has not regulated or directly negotiated drug prices, leaving prices largely set by the private market. These negotiations occur only after an exclusivity period following Food and Drug Administration (FDA) approval: currently, small molecule drugs become eligible after 9 years, while biologics are eligible after thirteen [1]. Critics argue that this longer protection for biologics, which are more expensive and target rare diseases, disincentivizes investment in small molecule drugs that are typically cheaper and serve broader populations.

The E.O. directs HHS Secretary Robert F. Kennedy Jr. to work with Congress to “end the distortion.” [2] E.O.s are instructions to the executive branch to implement policies, but do not have the force of law. Addressing this issue would ultimately require legislative action, as modifying the current incentive structure for pharmaceutical investment falls within Congress's authority. Although no legislation has yet enacted these changes, the E.O. signals a major policy shift with potentially serious consequences.

If small molecule drugs were granted the same 13‐year exemption as biologics, more than half of the drugs selected for Medicare negotiation—representing $61 billion USD in Part D spending—would have been excluded. These include Eliquis, Jardiance, Farxiga, Entresto, and Ozempic/Rybelsus/Wegovy [3]. In dermatology, many patients rely on small molecule therapies for chronic inflammatory diseases. This includes not only systemic oral medications but also topical small molecules, which play a critical role in dermatologic care. Extending the timeline under the guise of supporting innovation may instead limit affordability and access for patients while increasing government spending.

For example, extending the small molecule negotiation window from 9 to 13 years could cost Medicare an estimated $350 million to $2.1 billion USD in lost savings across just the three most expensive drugs in dermatology (Table 1).

**TABLE 1:** Calculations for three brand‐name small molecule drugs with patent approval within the past 9 years and annual Medicare Part D spending greater than 200 million, thus meeting eligibility criteria for negotiation.

Brand name	Generic	Total beneficiaries in 2022	Total Medicare spending with generic availability (status quo) over 4 years	Total Medicare spending over 4 years with delayed expiry	Projected increase in Medicare spending
Otezla	Apremilast	24,076	$1,988,193,645.20	$2,840,276,636.00	$852,082,990.80
Xeljanz XR	Tofacinib citrate	13,973	$1,655,101,898.80	$2,364,431,284.00	$709,329,385.20
Rinvoq	Upadacitinib	15,674	$1,741,904,967.60	$2,488,435,668.00	$746,530,700.40

*Note:* Data were extracted from the 2022 Medicare Part D Dashboard and used to model projected increases in Medicare spending under a delayed‐negotiation timeline. Estimates assume a conservative 30% reduction in Medicare spending following negotiation.

While many of these small molecule drugs are not yet scheduled for negotiation, the policy shift could affect future candidates by allowing high‐cost therapies to stay at full price longer, delaying access to more affordable alternatives and exacerbating the financial burden on older adults. The resulting out‐of‐pocket costs from prolonged ineligibility for negotiation could exacerbate health disparities and undermine treatment adherence. Moreover, delaying negotiation until year 13 would leave Medicare with only a narrow window to realize savings before generic competition naturally lowers prices, undermining the core intent of the IRA.

Supporters of the policy argue the current 9‐year timeline discourages small molecule development and shifts investment toward biologics [4]. However, many small molecule drugs remain highly profitable within existing exclusivity periods, and evidence that shorter timelines deter innovation is mixed [5]. Any change would require congressional action, as the IRA's provisions cannot be amended by executive order alone.

Rather than extending protections for small molecule drugs, policymakers seeking parity could consider shortening the biologic negotiation window. This approach would promote equity, maximize savings, and expand access to affordable treatments. As Congress considers future reforms to the IRA, preserving mechanisms that improve access and control costs while supporting innovation is essential.

## Conflicts of Interest

N.J.P. and L.A.F. are employees of Rubrum Advising. J.S.B. is a consultant for Honeydew Care and Dexcel Pharma.

## Data Availability

The data that support the findings of this study are available in Medicare Part D Drug Spending Dashboard at https://data.cms.gov/tools/medicare‐part‐d‐drug‐spending‐dashboard. These data were derived from the following resources available in the public domain: Medicare Part D Drug Spending Dashboard, https://data.cms.gov/tools/medicare‐part‐d‐drug‐spending‐dashboard.
